# Combined Diagnostic Value of Hsa-miR-592 and Hsa-miR-9-3p in Plasma for Methamphetamine Addicts

**DOI:** 10.3390/ijms25168952

**Published:** 2024-08-16

**Authors:** Wenbo Li, Diandian Liu, Xiaokun Liu, Yun Lu, Ludi Zhang, Feng Yu, Hailei Yu, Chunling Ma, Bin Cong, Di Wen, Bing Xie

**Affiliations:** 1 Hebei Key Laboratory of Forensic Medicine, Collaborative Innovation Center of Forensic Medical Molecular Identification, Research Unit of Digestive Tract Microecosystem Pharmacology and Toxicology, College of Forensic Medicine, Hebei Medical University, Chinese Academy of Medical Sciences, Shijiazhuang 050017, China; wvnboo@163.com (W.L.); liudiandian2021@163.com (D.L.); liuxiaokun1997@163.com (X.L.); 15226523170@163.com (Y.L.); zldkid@hotmail.com (L.Z.); 18000714@hebmu.edu.cn (F.Y.); 18232587910@163.com (H.Y.); chunlingma@hebmu.edu.cn (C.M.); cong6406@hebmu.edu.cn (B.C.); 2 Key Laboratory of Neural and Vascular Biology, Ministry of Education, Shijiazhuang 050017, China

**Keywords:** METH, hsa-miR-592, hsa-miR-9-3p, ROC, BDNF

## Abstract

A number of studies have reported that drug addiction is associated with microRNAs (miRNAs). However, the roles of plasma miRNAs in methamphetamine (METH) addicts have not been clearly explained. This study aimed to profile a panel of miRNAs as non-invasive predictive biomarkers and therapeutic targets for METH addiction. Differentially expressed miRNAs were derived from next-generation sequencing technology (NGS) and were validated by quantitative real-time PCR (RT-qPCR). The diagnostic value of specific altered miRNAs was evaluated by receiver operating characteristic (ROC) analysis and area under the curve (AUC). NGS results revealed that 63 miRNAs were significantly altered in the METH-exposed paradigm. The levels of hsa-miR-592, hsa-miR-9-3p, hsa-miR-206 and hsa-let-7b-3p were significantly elevated in the plasma of METH addicts. Hsa-miR-9-3p was a useful biomarker discriminating METH addicts from normal (AUC was 0.756). Importantly, combining detection of hsa-miR-592 and hsa-miR-9-3p achieved the highest AUC of 0.87, with a sensitivity and specificity of 82.7% and 78.9%, respectively. Target gene BDNF decreased significantly in METH addicts. Although METH addicts showed significant depressive symptoms, there was no correlation between the expression level of miR-592 and miR-9-3p and the degree of depression. Our findings suggested that hsa-miR-592, hsa-miR-9-3p, hsa-miR-206, and hsa-let-7b-3p may play a potential role in the pathology of METH addiction, and a combination of hsa-miR-592 and hsa-miR-9-3p could serve as potential peripheral biomarker and therapeutic target for METH addiction.

## 1. Introduction

METH is a particularly addictive stimulant that affects the central nervous system (CNS) and belongs to the amphetamine family [1]. The World Drug Report 2023 showed that METH is probably the most widely supplied and utilized synthetic drug worldwide, which could exacerbate the global drug addiction crisis as the number of products and users rises [2]. METH addiction is characterized by recurrent cycles of drug use, abstinence and relapse. Chronic exposure to METH causes several psychosis-like behaviors, including hallucinations, paranoia, violence, suicidality, and mood disorders [3], while abrupt METH cessation could contribute to tiredness, depression, and aggressive behavior [4], which will have serious health and social effects. The nucleus accumbens (NAc) is a key component of the neural processes regulating impulsivity. Mechanistically, many studies found that methamphetamine addiction was often associated with increased extracellular dopamine levels in the NAc [5]. METH and its metabolism products can be detected in fluids of the body like saliva, blood, and urine [6] and are accumulated in hair [7], which are conventional means for identification of METH addicts at present. But body fluids and hair have strong timeliness and are highly susceptible to contamination, which makes it difficult to identify METH addicts. Even more serious is that there is no approved pharmacotherapy to treat METH addiction. Currently, METH addiction is treated with a combination of cognitive and behavioral therapies, but these traditional approaches suffer from high relapse rates [8]. Therefore, it is urgent to search for effective indicators for METH detection and remedy.

MiRNAs are little RNA molecules, approximately 22 nt in length, that control gene expression after the process of transcription. They have a significant impact on several physiological processes [9]. MiRNAs are active in the cell and also can dissociate outside the cell and exist stably in plasma or serum, which is called circulating miRNAs. Studies have shown that circulating miRNAs have characteristics of good stability, high specificities, easy detection, and dynamic changes during the development of disease [10]. In consequence, circulating miRNAs are ideal candidates as potential disease biomarkers and therapeutic targets. Numerous studies have shown that circulating miRNAs will be altered specifically in cancer [11], cardiovascular diseases [12], neurological diseases [13], substance use disorder [14], etc. A number of phase I clinical trials have indicated that an inhibitor targeting miRNAs has excellent safety and is a promising biological regulator [15,16] Our previous study found that miR-206 and miR-592 play an important role in the process of drug addiction, including evoking cocaine-associated memory and inhibiting opioid craving [17,18]. Although circulating miRNAs had great promise as a non-invasive diagnostic marker and therapeutic targets, the circulating miRNAs expression in the plasma of METH addicts remains to be improved, and attempts to use circulating miRNAs to identify METH addicts have not yet been established.

In the current study, a global profiling of miRNA expression relating METH was performed using NGS and RT-qPCR, and the diagnostic value of miRNAs was evaluated by ROC. Results demonstrated that a combination of hsa-miR-592 and hsa-miR-9-3p can clearly discriminate METH addicts from normal. Our results might provide a good foundation for the development of plasma miRNA candidates as biomarkers and potential therapeutic targets for METH addiction.

## 2. Results

### 2.1. miRNAs Expression Profiles in METH-Induced Conditioned Place Preference (CPP) Paradigm

**Figure 1:**
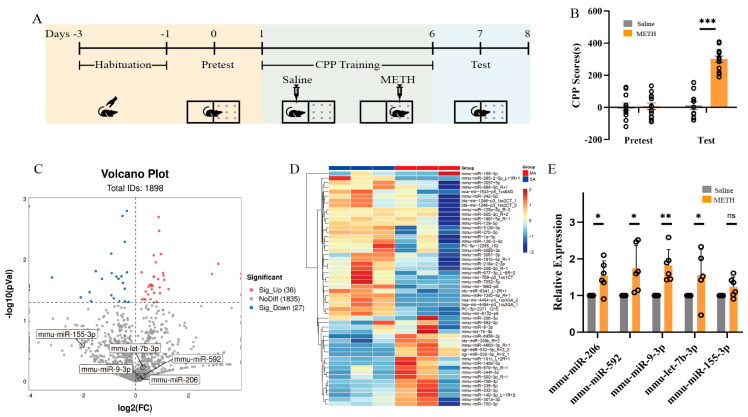
miRNAs expression profiles in METH-induced CPP paradigm. (**A**) The timeline of CPP training. (**B**) CPP scores of METH-induced mice elevated significantly at test in contrast to pretest (*n* = 13). (**C,D**) Heat mapping and volcano plots of the differential miRNAs in the NAc at pretest and test (*n* = 3). (**E**) The expression levels of mmu-miR-206, mmu-miR-9-3p, mmu-miR-592, mmu-let-7b-3p and mmu-miR-155-3p in the NAc on pretest and test (*n* = 6). * *p* < 0.05, ** *p* < 0.01, *** *p* < 0.001 and ns, not significant.

Differentially expressed miRNAs, respectively, derived from previous research, NCBI Gene Expression Omnibus (GEO) database (Supplementary Figure S1, Supplementary Table S1) and next-generation sequencing technology, including mmu-miR-9-3p, mmu-miR-592–3p, mmu-miR-206, mmu-let-7b-3p, and mmu-miR-155-3p. In order to generate the METH-induced CPP model, forty mice were distributed into two groups. One group underwent an intraperitoneal injection of saline, while the other group received an injection of METH at a dose of 1 mg/kg. The mice were then assessed for CPP scores on both pretest and test (Figure 1A). The statistical investigation, specifically a two-way ANOVA, exhibited a significant interaction impact [F (1, 44) = 44.39, *p* < 0.01] between the interventions (Saline vs. METH) and the outcomes (pretest vs. test). Statistically significant variations were seen between saline and METH-induced CPP mice [F (1, 44) = 45.73, *p* < 0.01]. Additionally, significant variations were discovered among the pretest and test groups [F (1, 44) = 47.86, *p* < 0.01] (Figure 1B). To verify the aforementioned miRNAs expression between the METH-induced CPP mice and saline group, miRNAs expression profile from the NAc tissue of mice was sequenced utilizing RNA-seq. A miRNA was considered significantly up- or downregulated if it exhibited a *p*-value less than 0.05, as determined by employing the negative binomial distribution method to compare the METH and saline groups. According to these criteria, the levels of 36 miRNAs were elevated, while the 27 miRNA levels were reduced in the METH group in contrast to the saline group (Figure 1C,D). The change tendency of mmu-miR-9-3p, mmu-miR-592-3p, mmu-miR-206 and mmu-let-7b-3p was consistent with the sequencing results. We found that mmu-miR-9-3p, mmu-miR-206, mmu-miR-592, and mmu-let-7b-3p were significantly upregulated by RT-qPCR, *p* < 0.05 (Figure 1E). The four miRNAs mentioned above enter into the follow-up experimental validation.

### 2.2. Participants Characteristics

A total of 81 METH addicts and 76 matched normal were enrolled in the present study. Table 1 provides a concise overview of their ages, gender, marital status, levels of education, behavior of drug abuse, and mental condition. Of the 81 METH addicts, 36 (44.4%) were male and 45 (56.6%) were female, and their average age was 34.37 years. Younger addicts who were under the age of 25 make up the majority of all patients with METH addicts, reaching 53.1%. The frequency of METH addicts using drugs was high; 42% of METH addicts use drugs on a daily basis. Furthermore, METH addicts had obvious craving for drugs during withdrawal (VAS = 4). Evaluation of neuropsychiatric symptoms showed that METH addicts were apparently depressed during withdrawal; the SDS score achieved was 53.25, *p* < 0.01.

### 2.3. Expression Levels of miRNAs in METH Addicts Plasma

**Figure 2:**
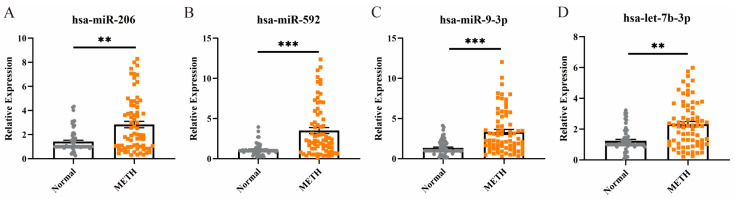
Plasma miRNAs relative expression level with normal and METH addicts. (**A**–**D**) Plasma hsa-miR-206, hsa-miR-592, hsa-miR-9-3p and hsa-let-7b-3p levels in the normal and METH addicts. The relative plasma miRNA expression levels were standardized cel-miR-39-3p. Every *p*-value was detected with a nonparametric Mann–Whitney test (** *p* < 0.01, *** *p* < 0.001).

The study objects were randomly divided into training (75%) and testing (25%) sets. The training set included 57 normal and 61 METH addicts, and the testing set included 19 normal and 20 METH addicts. Results from qPCR are shown in Table 2. In the training sets, compared with normal plasma samples, plasma hsa-miR-206, hsa-miR-592, hsa-miR-9-3p, and hsa-let-7b-3p levels were significantly elevated in METH addicts, *p* < 0.01. The findings obtained from the testing set exhibited an elevated degree of consistency with the outcomes seen in the training group. Furthermore, we performed a nonparametric Mann–Whitney test on the level of hsa-miR-592, hsa-miR-206, hsa-miR-9–3p, and hsa-let-7b-3p on the aggregate level, the result showed that the above miRNA expression levels were all increased (Figure 2A–D).

### 2.4. ROC Analysis

**Figure 3:**
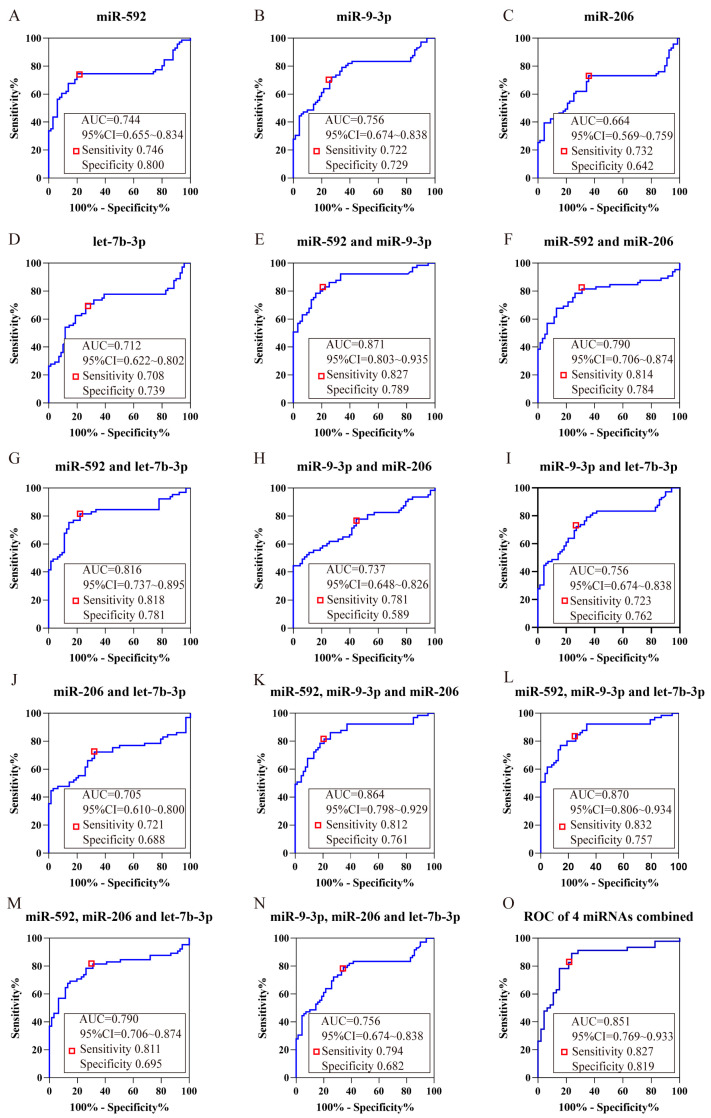
Receiver-operator characteristic (ROC) plot. (**A**–**D**) The ROC curve of a single miRNA. (**E**–**O**) The ROC curve of the combination of hsa-miR-206, hsa-miR-592, hsa-miR-9-3p and hsa-let-7b-3p. The identification accuracy of METH addicts was estimated utilizing the AUC. Maximum categorization accuracy is detected to occur at the point revealed by the red box. The sensitivity and specificity are demonstrated at the point when their combined value reaches its maximum.

To further evaluate the discriminatory capability of aforesaid miRNAs for the normal and METH addicts, we drew the ROC curve and determined the area under the curve (AUC). The ROC curve analysis revealed that hsa-miR-9-3p exhibited the greatest AUC values, reaching 0.756 (95% CI, 0.674~0.838), with a sensitivity and specificity of 72.2% and 72.9%, respectively, for METH diagnosis at the best cut-off point, which the sum of sensitivity and specificity reaches the maximal value (Figure 3B). Moreover, a model of binary logistic regression was generated to evaluate the diagnostic value of the mixture of hsa-miR-206, hsa-miR-592, hsa-miR-9-3p and hsa-let-7b-3p including the combination of two, three and four miRNAs, and computed the AUC of the ROC curve. The highest AUC of mixed hsa-miR-592 and hsa-miR-9-3p achieved 0.871 (95% CI, 0.803~0.935), and the sensitivity and specificity of the best cut-off point were 82.7% and 78.9% (Figure 3E). Its preliminary results indicated that a combination of hsa-miR-592 and hsa-miR-9-3p had excellent ability to differentiate between the normal and METH addicts.

### 2.5. Identification of miRNAs Target Genes

**Figure 4:**
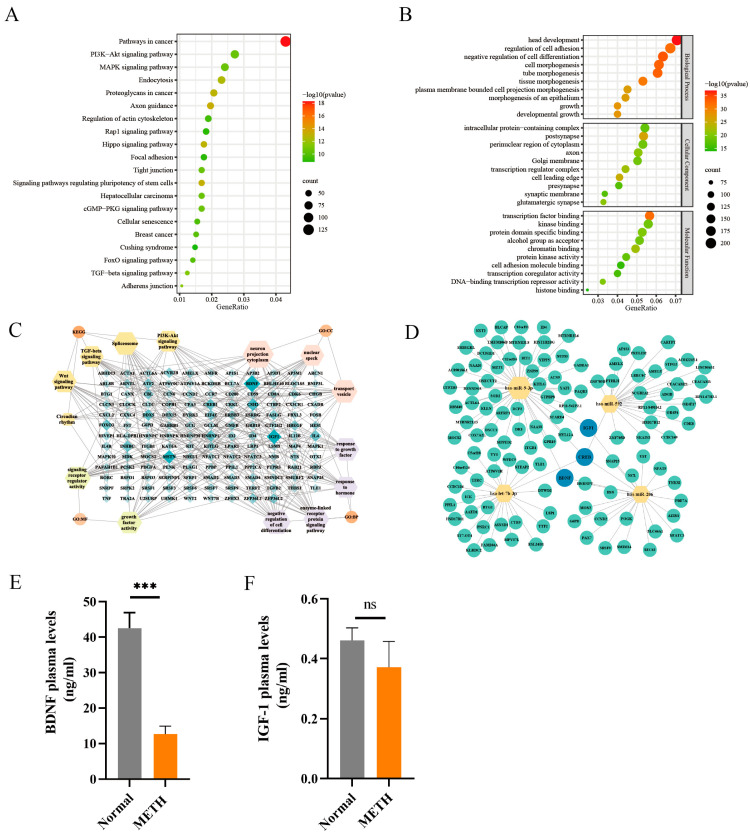
Identification of genes targets of miRNAs in the plasma of METH addicts. (**A**) Functional relationship of over-mutated miRNA genes with the KEGG pathway. The graph illustrates the 20 most enriched pathways (*Y*-axis) in the genes that encode proteins. The size of the dot denotes the quantity of genes that encode proteins, while the color of the dot denotes an adjusted *p*-value. (**B**) Upregulation miRNA GO analysis (BP, CC, MF) result. (**C**) MiRNA pathways and functional networks predicted by Cytoscape. The dimensions of a node are directly related to its closeness centrality. (**D**) Identification of gene targets of miRNAs deregulated in predicted genes. (**E**,**F**) Plasma levels of BDNF and IGF-1 in METH addicts and the normal. *** *p* < 0.001, ns denotes no significant difference.

To predict target genes of upregulated miRNAs in METH addicts, TargetScan and miRDB prediction websites were used to analyze federatively, 2904 relevant genes were screened out. We next analyzed KEGG pathways and Gene Ontology (GO) terms by using Metascope websites, the top 20 KEGG pathways and GO terms were shown (Figure 4A,B). These signaling pathways and functions are deeply related to the nervous systems. We found that 37 genes were enriched on the PI3K/AKT pathway. Moreover, we drew a network map between the genes and pathway and GO terms with Cytoscape v3.9.1 and found that BNDF, IGF-1, CREB1, MSTN, FOXO3, and INHBC involved multiple signaling pathways and GO terms (Figure 4C). In addition, we partially showed the network map between the genes and miRNAs (Figure 4D). The contents of secreted proteins BDNF and IGF-1 were analyzed in plasma by ELISA. We found that the level of BDNF in the plasma of METH addicts diminished significantly, *p* < 0.001 (Figure 4E), but the content of IGF-1 showed a nonsignificant change, with *p* = 0.11 (Figure 4F).

### 2.6. Correlation Analysis of Clinical Indicators

**Figure 5:**
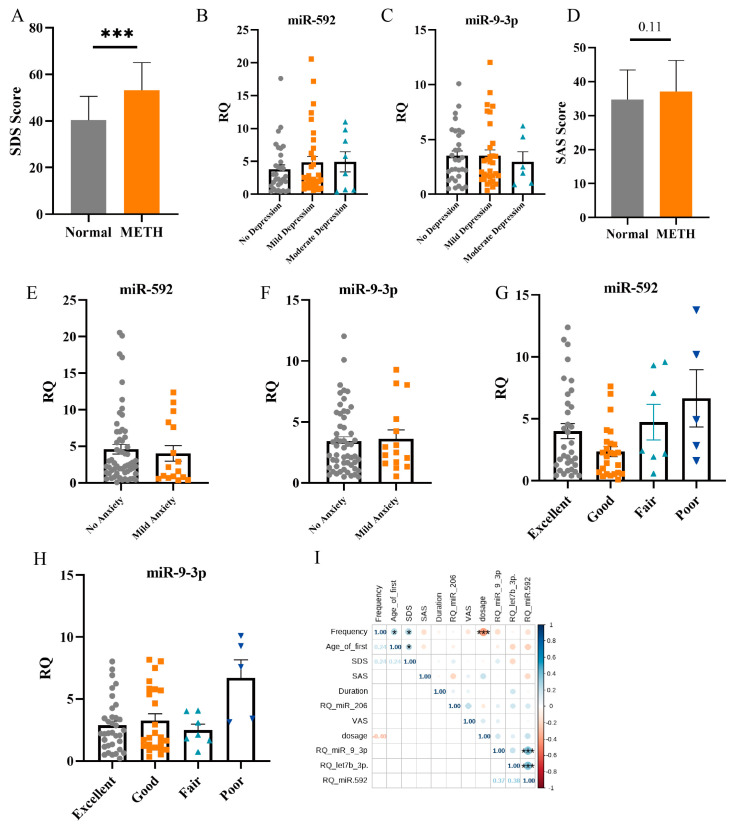
Correlation analysis of clinical indicators. (**A**) The SDS score of the normal and METH addicts. (**B**,**C**) The expression level of miR-592 and miR-9-3p in different degrees of depression. (**D**) The SAS score of the normal and METH addicts. (**E**,**F**) The expression level of miR-592 and miR-9-3p in different degrees of anxiety. (**G**,**H**) The expression level of miR-592 and miR-9-3p in different degrees of drug craving. (**I**) Heat map of r- and *p*-values for the Pearson correlation (*** *p* < 0.001, * *p* < 0.05).

The SDS score showed that METH addicts were apparently depressed during withdrawal (Figure 5A). The depression degrees of 81 METH addicts were separated into no (*n* = 33), mild (*n* = 37) or moderate (*n* = 11) grade groups in accordance with the World Health Organization classification. There were no appreciable group differences in miR-592 and miR-9-3p expression levels (Figure 5B,C). The SAS score suggested that anxiety degrees showed no obvious differences between the normal and METH addicts (Figure 5D). The expression levels of miR-592 and miR-9-3p did not display marked differences in no anxiety (*n* = 62) and mild anxiety (*n* = 16) groups (Figure 5E,F). The level of desire for drugs of METH addicts was separated into excellent (*n* = 37), good (*n* = 28), fair (*n* = 8) and poor (*n* = 5) grade groups referring to clinical pain grading. MiR-592 and miR-9-3p expression levels were elevated in the poor group (Figure 5G,H). The measured miRNAs and protein content were analyzed by the Pearson correlation with mental status and drug use. We found that the frequency and number of using the drug were negatively correlated in METH addicts (r = −0.4, *p* < 0.01), degree of depression was positively correlated with the frequency of using drugs and the age of first drug usage (r = 0.24, *p* < 0.05), and the plasma miR-592 expression level in METH addicts was positively connected with miR-9-3p and let-7b-3p (r = 0.37, *p* < 0.01) (Figure 5I).

## 3. Discussion

METH is a non-catecholamine and sympathomimetic stimulant [19], widely abused around the world, causing serious social and health problems. The World Drug Report 2023 shows METH use and trafficking are expanding and affecting more regions. It probably is the most widely used and supplied synthetic drug worldwide. Currently, METH has been classified as a controlled substance in most countries. It is generally recognized that detection of METH or its metabolites in body fluids or hair is the gold standard for METH detection [20,21]. However, the time window for body fluids detection is short and is highly susceptible to contamination. The effective detection period of METH is only 30 min to 72 h in saliva and blood. METH and its metabolites could almost be detected in urine beyond 7 days [22]. Although the effective detection period of hair is longer (1 to 6 months), it takes time to grow and is influenced by hair care factors [23]. There is a gap period for the detection of METH when it is completely metabolized in body fluids, and hair has not yet grown. Even more serious is that there is no approved pharmacotherapy to treat METH addiction. Psychotherapies, a routine treatment for psychological problems, has not been widely promoted due to poor long-term recovery and relapse [24,25]. Therefore, it is of great importance to find new approaches to improve the detection and therapeutics for METH addiction. This study has been devoted to finding novel plasma miRNA candidates as biomarkers and potential therapeutic targets for METH addiction.

Accumulating evidence reveals that circulating miRNAs are specifically expressed in multiple diseases and fulfill many characteristics of an ideal biomarker, including good stability, high specificities, easy detection, and dynamic changes during the development of disease [26]. For example, it has been proposed that seven plasma miRNAs in combination with CA-125 can improve early-stage ovarian cancer diagnosis [27,28]. In addition, the levels of certain miRNAs that circulate can be used to determine the severity of COVID-19 in individuals who are admitted to the hospital. Specifically, miR-148a-3p, miR-486-5p, and miR-451a have been shown to be linked to the need for ICU treatment [28]. Serum miR-206 and miR-132 have the potential to serve as markers for diagnosing MCI in individuals with neurological illnesses. Additionally, these biomarkers are connected with the Montreal Cognitive Assessment score for people with Alzheimer’s disease [29]. Moreover, investigation has found a group of irregular miRNAs in blood samples from individuals with epilepsy. This group includes elevated levels of miR-182-5p, miR-142-5p, miR-93-5p, and miR-199a-3p, as well as reduced levels of miR-574-3p [30]. In terms of drug addiction, prior work in our lab has shown that miR-592-3p can enhance morphine craving incubation by targeting TMEFF1 [31]. In a cross-sectional investigation, it was shown that the levels of miR-206, let-7b-5p, and miR-486-5p were consistently and significantly elevated in individuals who abuse heroin. Additionally, the miR-9-3p level was significantly greater in those who abuse METH in contrast to individuals who do not abuse any kind of drug [32]. However, it must be acknowledged that non-coding RNAs are more commonly used to study cancer. The small and heterogeneous sample size limited the power of this study. Furthermore, METH addict tissue samples are difficult to obtain and this imposed certain limitations on our study.

The present study revealed that the expression levels of miR-206, miR-592, miR-9-3p, and let-7b-3p are altered in the plasma of METH addicts, and the highest AUC of combining hsa-miR-592 and hsa-mi-9-3p achieved 0.871. MiR-206 is expressed in various tissues and diseases, like cancer, bone disease, neurological disease, cardiovascular disease, liver disease, and kidney disease. In our study, the diagnostic efficacy of miR-206 was not high; AUC was less than 0.7, which may be related to the low tissue specificity of miR-206 [33]. Research has shown that reduced levels of miR-592 inhibited the development of neurons. However, administering miR-592 as a therapy somewhat improved the autism-like phenotypes [34]. Let-7b-5p and miR-9-3p are two miRNAs that have been discovered earlier; there’s a lot of research on them. An investigation revealed that by suppressing the treatment of miR-331-3p and miR-9-5p, the progression of Alzheimer’s disease may be stopped, and this is achieved by enhancing the process of the autophagic clearance of amyloid beta [35]. A separate investigation discovered that there was a significant reduction in the expression of neuronal let-7b-5p, which was connected to the activation of the Hippo pathway. This activation was accompanied by a rise in the neuronal/nuclear ratio of Yes-Associated Protein (YAP), as well as an elevation in the production of cleaved caspase-3 in the neurons of neonatal encephalopathy [36]. In our study, miR-592 and mi-9-3p had good accuracy in the diagnosis of METH addiction. The reason for the better diagnostic efficacy of miR-592 and mi-9-3p could be that they have higher neural tissue specificity.

The present study attempted to explore the correlation between the toxicological profile of participants and miRNA levels. Although METH addicts showed obvious depression symptoms and METH craving, unfortunately there was no significant difference in the expression levels of plasma hsa-miR-9-3p and has-miR-592 among addicts with different clinical manifestations. In other words, hsa-miR-9-3p and has-miR-592 had little ability in discriminating the severity of METH addicts. Thus, in a subsequent study, we attempt to design more detailed grouping and to explore the differences of plasma miRNA expression in METH addicts with different degree of addiction, providing a clinical basis for follow-up treatment [37]. In addition, there was no significant correlation between the expression level of hsa-miR-9-3p, hsa-miR-592, hsa-miR-206, hsa-let7b-3p and the toxicological profile of METH addicts. This may be related to the fact that plasma miRNAs are affected by multiple systems [38].

The ROC curve, a preferred statistical method to evaluate the model, reflects the relationship between sensitivity and specificity and is generally used to judge whether a certain factor has diagnostic value for the diagnosis of diseases [39]. The AUC is a measure of prediction accuracy, ranging from 0 to 1. The closer the number is to 1, the greater the level of discrimination. The cut-off point is calculated by the Youden index. In this study, we used ROC curves to evaluate the plasma miRNA diagnostic performance. MiR-9-3p had the biggest AUC, but sensitivity and specificity were unsatisfactory, which may be related to the small amount of data. Predicted value probability from logistic regression was utilized to calculate the combined effect of miRNAs [40].

To further explore the mechanisms of dysregulated miRNAs, the relevant target genes were predicted, and the pathways of Gene Ontology (GO) and KEGG were analyzed. The outcomes indicate that the PI3K/AKT signaling pathway was enriched with the largest number of differential genes. The PI3K/AKT signaling pathway is an intracellular mechanism that facilitates the transduction of signals inside a cell, leading to various cellular processes such as metabolic processes, growth, cell survival, growth, and angiogenesis in response to signals from outside the cell [41]. The PI3K/AKT signaling pathway has shown significant involvement in METH addiction. An investigation has shown that the activation of metabotropic glutamate receptor 5, AKT/PI3K signaling, and the NF-κB pathway are all implicated in the METH-induced elevation of IL-6 and IL-8 expression in astrocytes. Different research discovered that aromadendrin has the ability to protect neurons against the neurotoxic effects caused by METH. This protection is achieved by modulating the endoplasmic reticulum stress and the PI3K/Akt/mTOR signaling pathway [42]. BDNF, a secretory protein, is widely distributed and intensively investigated in the mammalian brain [43], It plays a role in the PI3K/AKT signaling pathway. An investigation discovered that BDNF has an impact in mediating the arrangement of miR-132-5p, miR-218-5p, and miR-690 inside EVs produced from neurons [44]. In the present study, the BDNF content in the plasma of METH addicts reduced significantly, which may be bound to and silenced by miRNA at the level of transcription.

Several weaknesses should be noted. First, our investigation was performed utilizing a cross-sectional design that was unable to reveal the dynamic changes in plasma miRNAs in METH addiction and withdrawal and conduct longitudinal studies with larger sample sizes in the future. In addition, the sequences of human miRNA and mouse miRNA were not completely consistent, so we tried our best to ensure the sequence consistency of the seed region. Future studies will aim to study these mechanisms of the homologous miRNAs. Although several investigations have looked at miRNAs as possible markers, a lack of standardization has hindered their clinical application [45]. Moreover, relatively quantitative statistical methods are another difficulty for miRNA as biomarkers. Next-generation sequencing technology and absolute quantification may be new solutions.

## 4. Materials and Methods

### 4.1. Animals

This study employed male C57BL/6J mice weighing between 20 and 25 g, which were sourced from Beijing Vital River Laboratory Animal Technology Co., Ltd., Beijing, China. The animals were kept together in groups and provided with unrestricted utilization of food and water. Each animal was uniquely identified by ear punching. The housing conditions maintained a stable temperature of 21 ± 2 °C and humidity of 60–65%. The animals underwent a cycle of twelve hours of light and darkness, with lights turning on at 8:00 a.m. The animal investigations were performed in compliance with the NIH’s Guide for Care and Use of Laboratory Animals and the Animal Care and Use Committee of Hebei Medical University. A total of eighty mice were utilized in this investigation.

### 4.2. Drug

The amounts of METH (with a purity over 95%) provided by the Public Security Bureau of Beijing Municipality in Beijing, China, were adjusted to a suitable injection dosage of 1 mL per kilogram of body weight utilizing saline solution.

### 4.3. Conditioned Place Preference

METH addiction in mice was assessed utilizing the conditioned place preference (CPP) test [18]. The CPP device had two compartments that exhibited distinguished visual and tactile signals. These compartments were divided by a clapboard, which included a tiny door. The equipment was manufactured by JLBeh Soft-tech Co. Ltd. in Shanghai, China. CPP scores were identified as the period in the METH-paired chamber subtracted from the period in the saline-paired chamber in seconds. Briefly, CPP involves three stages: pretest, conditioning, and test. During the pretest (0 day), mice were given 15 min to freely explore, and the period they spent in the two side compartments was recorded. Any time spent in compartments with a CPP score of more than 150 s was removed. During the conditioning stage, mice underwent a treatment regimen for 6 days. They received alternating administrations of saline (1 mg/kg, i.p.) or METH (1 mg/kg, i.p.) every 6 h. Following each injection, the mice were restricted for 45 min to either the drug- or saline-paired compartment. In the test (7 day), mice were placed for 15 min in the CPP apparatus with no clapboard, and the period they spent in the two side compartments was recorded.

### 4.4. RNA Sequencing

The NAc tissues of the mice were immediately obtained following the CPP test, and the sample size was 3. The TruSeq Small RNA Sample Prep Kits (Illumina, San Diego, CA, USA) were utilized to construct sequence libraries, following the directions provided by Lian Chuan Biotech, Hangzhou, China. Small RNA-seq sequencing libraries underwent sequencing on Illumina HiSeq4000 sequencers, single-ended 50 bp. Finally, ACGT101-miR (LC Sciences, Houston, TX, USA) was employed to analyze the differential expression of miRNAs.

### 4.5. Participants

A total of 81 METH addicts, 18–60 years, were recruited consecutively from cross-sectional and longitudinal studies (ChiCTR2100045976) in the Compulsory Isolation Center for Drug Rehabilitation in Shanxi and Hebei, China, during 2019 to 2021. The inclusion criteria of the subjects were as follows: an age of 18–60 years old, meeting the ICD-10 diagnostic criteria for drug addiction throughout the physiological detoxification phase, METH intake more than once at least. Exclusion criteria of all subjects included the following: any physical, neurological or psychiatric condition, users of other drugs or mixed drugs. All the participants were invited to participate in the questionnaire. Depression, anxiety, and craving for drugs were assessed by self-rating depression scale (SDS), visual analog scale (VAS), and self-rating anxiety scale (SAS). A total of 76 healthy controls were randomly selected from medical examination centers and were frequency-matched for age and gender to the cases. The investigation received authorization from the ethical council of Hebei Medical University, and all patients provided written informed permission.

### 4.6. Plasma Collection

A venous blood sample (2 mL) was collected from each participant in EDTA-anticoagulant tubes after 12 h of overnight fasting. The blood samples were left for 30 min at room temperature before being centrifuged for 10 min (3000 r, 4 °C). The upper aliquot solution (1 mL) of plasma samples was transferred to clean 1.5 mL RNase-free centrifuge tubes and immediately kept at −80 °C.

### 4.7. Quantitative Real-Time PCR

Total RNA was obtained from a sample of 300 μL plasma utilizing TRIzol LS Reagent (Thermo Fisher Scientific, Waltham, MA, USA). All-in-One™ miRNA First-Strand cDNA Synthesis Kit 2.0 (Gene Copoeia, Guangzhou, China) was used to reverse-transcribe into cDNA from 500 ng RNA. All qRT-PCR was carried out on the QuantSudio 7 Flex System (Applied Biosystems, Waltham, MA, USA) using the All-in-One™ miRNA qRT-PCR Detection Kit (Gene Copoeia, Guangzhou, China). Synthetic cel-miR-39 (RiboBio, Guangzhou, China) was selected as the internal reference. Relative expression was calculated using the 2^−∆∆Ct^ method.

### 4.8. Bioinformatics Analysis

TargetScan (targetscan.org/vert_80/) and miRDB (miRDB—MicroRNA Target Prediction Database) were employed to predict the miRNA target genes. Gene Ontology (GO) term enrichment and KEGG enrichment were performed using Metascape [46]. Moreover, we drew a network map between the genes and pathway and GO terms by Cytoscape v3.9.1 [47].

### 4.9. ELISA

Plasma BDNF and IGF-1 concentrations were measured separately by using the double antibody sandwich method ELISA Kits (ABclonal Technology Co., Ltd., Wuhan, China). Briefly, plasma samples were allowed to incubate for 2 h onto microplate wells that were precoated with the capture antibody. A detection antibody was then added to form a sandwich complex and streptavidin-HRP was added to bound the sandwich complex. After sufficient washing, the microplate wells were incubated with TMB substrate for 15 min, and the reaction was stopped with TMB-Stop solution, immediately read absorbance at 450 nm. The absorbance was plotted against the concentrations of the provided standards to generate the standard curve. The concentration values of every specimen were then determined employing the equation of the curve.

### 4.10. Statistical Analysis

SPSS 26.0 (Chicago, IL, USA) and GraphPad Prism 8 (San Diego, CA, USA) were utilized to conduct the statistical analyses. Differences between the 2 groups were tested employing Student’s *t*-test or Wilcoxon signed-rank test. One-way ANOVA or two-way ANOVA were conducted for grouped or multivariate analysis. The ROC curve was generated, and AUC-ROC was calculated to assess the diagnostic efficacy of miRNAs assay. The correlation was compared using the Pearson correlation coefficient. Outcomes are expressed as the mean ± SEM; a *p*-value of < 0.05 was considered statistically significant.

## 5. Conclusions

In summary, the current study exhibited that hsa-miR-206, hsa-miR-592, hsa-miR-9-3p and hsa-let-7b-3p were significantly raised in the plasma of METH addicts. A combination of Hsa-miR-592 and hsa-miR-9-3p had excellent diagnostic value to differentiate between normal and METH addicts, and BDNF may be negatively regulated by hsa-miR-592 and hsa-miR-9-3p—they may serve as a potential biomarker for identifying METH addiction. Regrettably, hsa-miR-592 and hsa-miR-9-3p expression levels showed no other clear correlation with the clinical symptoms of METH addicts. More samples and more detailed clinical data are needed in the future.

## Figures and Tables

**Table 1:** Human subject features ^a^.

Variable	Normal	METH	*p*-Value ^b^
	*n* = 76	*n* = 81	
Age, years	34.01 ± 4.99	34.37 ± 7.42	0.457
Sex (%)			0.528 ^c^
Male	30 (39.5)	36 (44.4)	
Female	46 (60.5)	45 (56.6)	
Marital status (%)			
Married	_	22 (27.2)	
Single	_	34 (42)	
Divorced	_	25 (30.8)	
Educational degree (%)			
Elementary school	_	22 (27.2)	
High school	_	54 (66.7)	
University	_	5 (6.2)	
Age of first drug use (%)			
Less than 18	_	15 (18.5)	
18~25	_	28 (34.6)	
Greater than 25	_	38 (46.9)	
Frequency of drug use			
Once a day		34 (42)	
Two to six a week		21 (25.9)	
Once a week		11 (13.6)	
Every few weeks		15 (18.4)	
Duration of drug use			
Several days		20 (24.7)	
Several weeks		25 (30.9)	
Several years		36 (44.5)	
Profession			
Exist	none	15 (18.5)	
None	none	66 (81.5)	
Clinical indicators			
VAS	_	4	
SAS	34.76	37.11	0.102
SDS	40.36	53.25	<0.01

^a^ Data are presented as the mean ± SD. ^b^ METH addict group vs. normal group. ^c^ Chi-squared test.

**Table 2:** Relative expression levels of four plasma miRNAs in the training and test sets ^a^.

miRNAs	Training Set		
	Normal (*n* = 57)	METH (*n* = 61)	*p*-Value ^b^
hsa-miR-206	1.59 ± 1.26	4.77 ± 7.1	0.0004
hsa-miR-592	1.47 ± 1.39	6.56 ± 9.89	0.0001
hsa-miR-9-3p	1.66 ± 2.17	4.74 ± 6.32	0.001
hsa-let-7b-3p	1.39 ± 0.88	3.00 ± 3.05	0.0002
	Testing set		
	Normal (*n* = 19)	METH (*n* = 20)	*p*-value ^b^
hsa-miR-206	1.58 ± 1.61	3.84 ± 3.53	0.006
hsa-miR-592	1.50 ± 2.10	5.66 ± 8.00	0.014
hsa-miR-9-3p	1.77 ± 1.75	5.25 ± 7.43	0.025
hsa-let-7b-3p	1.23 ± 0.81	2.33 ± 2.15	0.043

^a^ Dates are presented as the mean ± SD. ^b^ Normal group vs. METH group.

## Data Availability

Data is contained within the article and Supplementary Materials.

## Supplementary Materials

The following supporting information can be downloaded at: https://www.mdpi.com/article/10.3390/ijms25168952/s1.
