# Supplementary material for: Combined Diagnostic Value of Hsa-miR-592 and Hsa-miR-9-3p in Plasma for Methamphetamine Addicts
Source: Int J Mol Sci. 2024 Aug 16;25(16):8952. doi: 10.3390/ijms25168952 (PMC11354292; doi:10.3390/ijms25168952)
Supplement: Supplementary file 1 [file ijms-25-08952-s001.zip › ijms-3098346-supplementary.pdf]

Supplementary Material

Supplementary Table S1. Differentially Expressed miRNAs Related to Drug Addiction

previous research	GEO DataSets		
	GSE106314	GSE41952	GSE42506
miR-206[1]	miR-9-3p	miR-9-3p	miR-155-3p
miR-592[2]	Let-7b-3p		

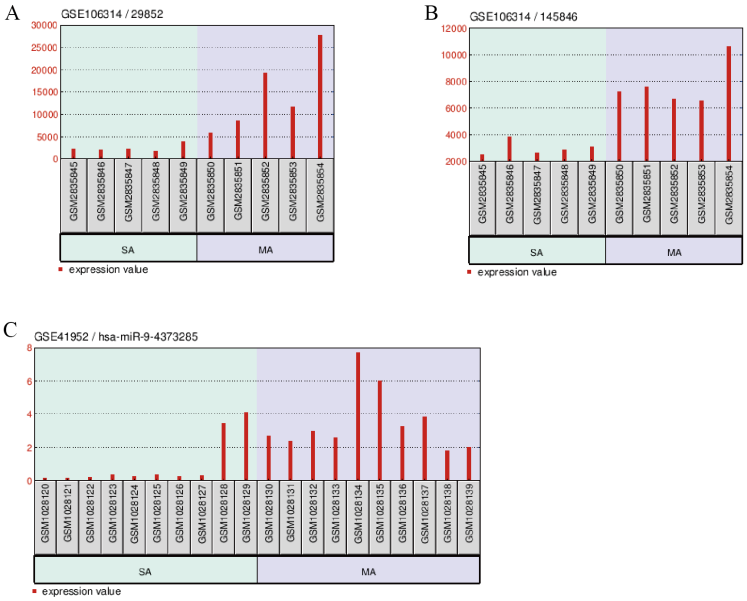

Supplementary Figure S1. Differentially expressed miRNAs related to drug addiction from GEO DataSets. (A)miR-9-3p expression was significantly elevated in the GSE106314. (B) let-7b-3p expression was significantly elevated in the GSE106314. (C) miR-9-3p expression was significantly elevated in the GSE41952.

1. Shen, Q.; Xie, B.; Galaj, E.; Yu, H.; Li, X.; Lu, Y.; Zhang, M.; Wen, D.; Ma, C., CircTmeff-1 in the nucleus accumbens regulates the reconsolidation of cocaine-associated memory. *Brain Res Bull* **2022,** 185, 64-73.https://doi.org/10.1016/j.brainresbull.2022.04.010
2. Xie, B.; Zhang, J.; Ma, C.; Yu, H.; Ni, Z.; Cong, B.; Wen, D., Roles of miR-592-3p and Its Target Gene, TMEFF1, in the Nucleus Accumbens During Incubation of Morphine Craving. *Int J Neuropsychopharmacol* **2022,** 25, (5), 412-424.https://doi.org/10.1093/ijnp/pyac004
